# Implementation of a Best Practice Advisory–Based HIV Screening Program in the Emergency Department with Integrated Infectious Diseases: A Descriptive Study

**DOI:** 10.1093/ofid/ofag475

**Published:** 2026-08-07

**Authors:** Brianna Hohmann, Madison Patrus, Smitha Gudipati, Thomas Hagerman, Nicholas Yared, Dalton Bender, Shannon Payne, Jun Jin, Jacob Manteuffel, Indira Brar

**Affiliations:** Department of Medicine, Michigan State University, East Lansing, Michigan, USA; Division of Infectious Diseases, Department of Medicine, Henry Ford Health, Detroit, Michigan, USA; Department of Internal Medicine, Henry Ford Health, Detroit, Michigan, USA; Department of Medicine, Michigan State University, East Lansing, Michigan, USA; Division of Infectious Diseases, Department of Medicine, Henry Ford Health, Detroit, Michigan, USA; Department of Emergency Medicine, Henry Ford Health, Detroit, Michigan, USA; Department of Medicine, Michigan State University, East Lansing, Michigan, USA; Division of Infectious Diseases, Department of Medicine, Henry Ford Health, Detroit, Michigan, USA; Division of Infectious Diseases, Department of Medicine, Henry Ford Health, Detroit, Michigan, USA; Division of Infectious Diseases, Department of Medicine, Henry Ford Health, Detroit, Michigan, USA; Department of Public Health Sciences, Henry Ford Health, Detroit, Michigan, USA; Department of Emergency Medicine, Henry Ford Health, Detroit, Michigan, USA; Department of Medicine, Michigan State University, East Lansing, Michigan, USA; Division of Infectious Diseases, Department of Medicine, Henry Ford Health, Detroit, Michigan, USA

**Keywords:** best practice advisory, ED screening, HIV screening, linkage to care

## Abstract

**Background:**

Despite national guidelines recommending universal HIV screening, opportunities for testing are frequently missed in the emergency department (ED), a setting many vulnerable populations rely on for primary care. We describe a collaborative ED-based program created to reduce barriers to HIV screening.

**Methods:**

This was a retrospective, observational study describing a best practice alert (BPA)-based opt-out HIV screening program established in an ED in Detroit, Michigan, in 2019. A key program efficiency feature was collaboration between ED and infectious disease (ID) teams. Medical records for patients who had a newly diagnosed HIV infection from a screening test during an ED visit between 2019 and 2025 were analyzed. Demographic and clinicopathologic features were described. Primary outcomes included annual rates of newly diagnosed HIV infections, linkage to care, time to initial clinical visit and antiretroviral therapy, and posttreatment viral suppression.

**Results:**

Of 75 914 patients screened for HIV after program implementation, 176 (0.23%) were newly diagnosed with HIV. Almost 60% of newly diagnosed patients were linked to medical care (n = 104) within 90 days (median, 13 days). Median time to antiretroviral treatment initiation was 9 days, and 54 patients (72.9%) showed evidence of viral suppression at 12 months.

**Conclusions:**

Through close collaboration between ED and ID teams, a BPA system for ED HIV screening can remove structural barriers and reach patients with limited access to care, advancing equitable access to diagnosis, linkage to care, and HIV treatment.

## Teaser

An opt-out, best practice alert–based HIV screening program in the emergency department (ED) reduced barriers to HIV testing for vulnerable populations. Close collaboration between ED and infectious disease teams allowed rapid linkage to care and HIV treatment initiation in previously undiagnosed patients.

The United States Preventive Services Task Force recommends a one-time HIV screening for all individuals aged 15–65 years [1]. Since 2006, the Centers for Disease Control and Prevention (CDC) has further recommended universal HIV screening in all healthcare settings, including emergency departments (EDs), regardless of patient-reported or perceived risk [1], which is a strategy reinforced by the Ending the HIV Epidemic initiative [2]. Despite these national recommendations for universal screening, routine HIV testing remains inconsistently implemented and missed opportunities are common, particularly among vulnerable populations for whom the ED serves as a critical resource for HIV and other sexually transmitted infection (STI) screening.

Multiple barriers limit the routine adoption of HIV testing in the ED, including perceived lack of time, unfamiliarity with screening guidelines, clinician discomfort with delivering positive results, concerns regarding follow-up, and misperceptions of patient risk [3]. Workflow-integrated systems that facilitate collaboration between emergency medicine and infectious diseases (IDs) may help overcome these challenges by streamlining testing, improving linkage to care, and enabling rapid initiation of antiretroviral therapy (ART), a cornerstone of strategies aimed at ending the HIV epidemic [4]. Early HIV detection is essential, as timely ART initiation is associated with improved retention in care, faster viral suppression, reduced transmission, and improved clinical outcomes for people with HIV [5, 6].

The ID Division at Henry Ford Health in urban Southeast Michigan has provided counseling, testing, and referral (CTR) services to individuals in the ED since 2006, initially emphasizing point-of-care rapid HIV testing for individuals who were deemed to be at increased risk of HIV infection. This targeted strategy focused on patients who were evaluated for STIs and other conditions associated with HIV exposure in the ED. However, testing volume remained limited, as CTR data from 2016 showed only 974 point-of-care HIV tests were performed in the ED, yielding 10 confirmed diagnoses (positivity rate 1%).

Therefore, in 2019, our institution transitioned to a nontargeted, opt-out HIV screening program aligned with CDC recommendations for universal screening in all healthcare settings, including EDs, regardless of perceived risk [1]. This transition was enabled by an amendment to the Michigan Public Health Code that eliminated the requirement for written consent while preserving patient notification and the opportunity to decline testing. The CTR program evolved from a point-of-care testing model to a best practice advisory (BPA)–driven electronic screening algorithm embedded within ED workflows, substantially expanding the scope of testing. The program also established secure, real-time communication between ED and ID teams to coordinate patient care and facilitate rapid linkage to treatment. Reactive HIV tests ordered in the ED automatically triggered confirmatory HIV 1/2 antibody testing, after which ID clinicians promptly disclosed results and arranged initial care visits. As an intentional component of the BPA pathway, the ID team maintained proactive patient engagement to streamline care coordination before the first outpatient ID appointment.

The objective of this study was to comprehensively describe our BPA-driven HIV screening system in the ED and evaluate clinical linkage to care outcomes among patients who tested positive for HIV. We also examined the demographic features, testing indications, and clinical characteristics among those who were newly diagnosed with HIV infection. Overall, this study provides essential insight for the development of effective ED-based HIV screening approaches aimed at improving identification of previously undiagnosed HIV infections and facilitating timely linkage to care.

## METHODS

### Study Design and Participants

**Figure 1.**
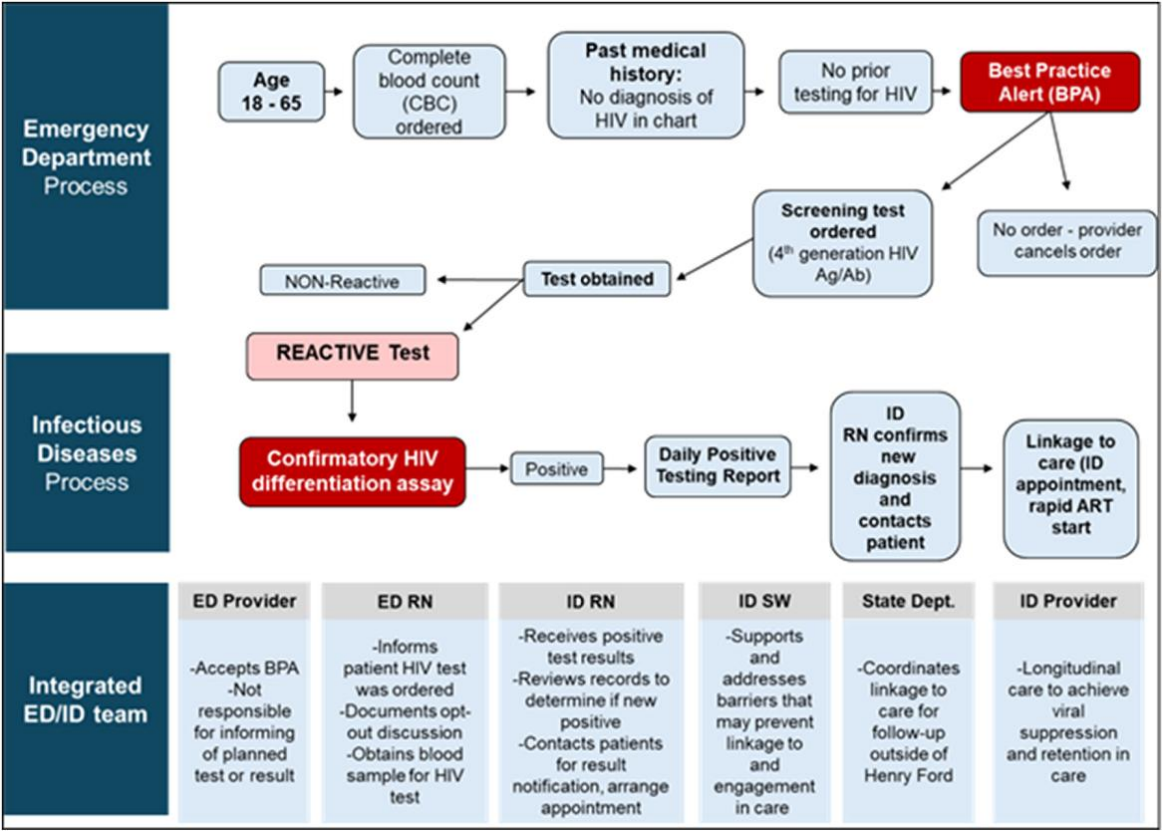
BPA workflow in the Henry Ford Emergency Department. Flow chart outlining the nontargeted ED HIV screening BPA algorithm. First steps begin in the ED for all adults receiving a complete blood count test ordered. Positive initial HIV screening test results trigger a confirmatory HIV differentiation test. Positive confirmatory results trigger patient contact and linkage to care. Bottom panels outline the roles of various team members in the process. Abbreviations: Ag/Ab, antigen/antibody; ART, antiretroviral therapy; BPA, best practice alert; ED, emergency department; ID, infectious disease; RN, registered nurse; SW, social worker.

This study was a retrospective, observational medical chart review following the implementation of a BPA-based system designed to increase HIV screening rate in a single urban ED in Detroit, Michigan, with ∼95 000 annual visits (Figure 1). Medical records from November 2019 to October 2025 were extracted. All people with newly diagnosed HIV infection within the study period were included, regardless of whether testing was prompted by the BPA or due to other factors (pregnancy, STI diagnosis, opportunistic infection, or other high-risk features). This project was approved by the Henry Ford Health Institutional Review Board (#16606-01). Informed consent was waived by the Institutional Review Board as this was a retrospective chart review.

### Intervention and Outcomes

The BPA-based system prompted a fourth generation HIV test for all individuals aged 18–65 years who had a complete blood count test performed in the ED, had no documented history of HIV infection, and had no prior HIV test available in the current electronic medical record (EMR), which was implemented in 2012. After HIV tests were ordered, nurses received a prompt to inform the patient of HIV testing and offer them the option to decline. Positive HIV test results automatically went through confirmatory testing with an HIV differentiation assay. Patients with positive confirmatory tests were contacted by the ID nurse, who discussed the diagnosis with the patient, offered counseling, scheduled a first appointment with ID, and engaged social work, if needed, to address any potential barriers to care. Patients who were unable to be contacted were referred to Michigan Department of Health and Human Services disease intervention specialists for further outreach.

The following data were extracted from the EMR: demographics, comorbid conditions, prior and current STI diagnoses, reason for HIV testing in the ED, and history of HIV testing. One ID physician performed chart abstraction using an electronic data entry form, and a second abstractor reviewed 30% of charts. Reasons for HIV testing were obtained through chart review by the ID physician and were based on the ED diagnosis and the medical decision-making section of the ED provider medical record note. HIV diagnoses were recorded as BPA only if, based on chart review, the ED provider was not already considering diagnoses, such as STI, opportunistic infection, high-risk features, or pregnancy in their decision-making. Primary outcomes included number of patients linked to care, time to first appointment from time of diagnosis, days to ART initiation from diagnosis, baseline viral load and CD4 counts, and viral suppression at 3, 6, and 12 months after diagnosis. The outcomes were calculated among participants with available follow-up data at each time point; viral suppression was defined as HIV-1 RNA <30 copies/mL. Linkage to care was defined as attending the first appointment with an ID specialist after a new HIV diagnosis. Secondary outcomes included new HIV test-positivity rates per year, and rate of BPA use relative to other reasons for HIV testing.

### Assessments, Outcomes, and Statistical Analysis

Demographic and clinical characteristics were reported with counts and percentages for categorical variables and either mean and standard deviation or median and interquartile range (IQR) for continuous variables, as appropriate. Annual HIV test positivity was calculated as the number of new HIV-1 diagnoses divided by the total number of HIV tests performed each year in the study period (2019–2025). Year-specific test positivity was compared with the reference year 2020 by estimating test-positivity rate ratios. Rate ratios, 95% confidence intervals, and *P*-values were obtained with log-link models (Poisson regression with an offset for the number of tests and robust standard errors) equivalent to Wald inference on the log rate ratio for 2-group comparisons. For outcomes analyses, patients without documentation of a postdiagnostic ID visit, ART initiation, or viral suppression testing at Henry Ford Hospital were considered missing data and were not included. Two-sided *P*-value of <.05 was considered statistically significant; *P*-values were not adjusted for multiple comparisons.

## RESULTS

During the study period, 75 914 patients in the ED were screened for HIV, of which 176 patients were newly diagnosed with HIV infection, yielding an overall new test-positivity rate of 0.232%. The highest HIV positivity rate occurred in 2021, at 0.360%; yet positivity rates did not significantly differ between any year relative to the first full year of BPA system implementation in 2020. The rate of positive HIV diagnoses based on BPA-based testing alone varied by year but did not increase or decrease consistently over time. The BPA-based HIV testing uptake rate by physicians and healthcare providers in the ED was 72.4% over the course of the study. The provider opt-out rate for BPA-based HIV testing varied by year, increasing from 2019 to 2022 and then decreasing from 2023 to 2025 (Table 1).

**Table 1.** Annual Rate of Newly Diagnosed HIV Infections in the Emergency Department

Year	Total HIV Tests, No.	New Positive HIV, No.	New Infection Rate, %	Rate Ratio Versus 2020 (95% CI)	*P* Value^a^	BPA-Only Diagnoses, %	BPA Opt-Out, %
2019^b^	2746	11	0.401	na	na	70.0	18.2
2020	15 651	37	0.236	Reference	na	83.3	27.5
2021	8334	30	0.360	1.52 (.94–2.46)	.08	55.2	29.6
2022	10 068	28	0.278	1.18 (.72–1.92)	.52	70.4	35.3
2023	13 809	28	0.203	0.86 (.53–1.40)	.54	70.4	27.7
2024	14 809	28	0.189	0.80 (.49–1.31)	.37	59.3	24.4
2025^b^	10 497	14	0.133	0.56 (.31–1.04)	.06	76.9	20.9
2019–2025	75 914	176	0.232	…	…	66.5	27.6

Abbreviations: BPA, best practice alert; CI, confidence interval; na, not applicable.

^a^ *P* values from Pearson χ^2^ (no Yates correction), comparing each year to 2020.

^b^2019 was the program launch year and included 13 November to 31 December; year 2025 included 1 January to 31 October.

The median age of people with newly diagnosed HIV infection was 33 years (IQR, 27–44). Most patients were Black (n = 146; 83%) and male (n = 129; 73%). Just over one-third were heterosexual (n = 63; 35.8%). Regarding HIV risk factors, 61 patients (34.7%) were men who have sex with men (MSM), 59 (33.5%) were heterosexual with multiple partners, 16 (9.1%) reported intravenous drug use, and 28 (15.9%) were unhoused. Comorbidities were uncommon, with hypertension being the most common in 24 patients (13.6%). Of the newly diagnosed patients, 32 (18.2%) had a prior STI diagnosis, most commonly syphilis (n = 15; 8.5%) and gonorrhea (n = 14; 8%; Table 2).

**Table 2.** Demographic Characteristics of Patients with Newly Diagnosed HIV Infection Identified in the Emergency Department

Characteristic	Total (N = 176)
Age, y, median (IQR)	33 (27–44)
Race/ethnicity	
Black	146 (83)
White	14 (8)
Hispanic	9 (5.1)
Sex	
Male	129 (73.3)
Female	47 (26.7)
Gender identity	
Male	109 (61.9)
Female	42 (23.9)
Transgender female	3 (1.7)
Sexual orientation	
Gay	34 (19.3)
Heterosexual	63 (35.8)
Bisexual	20 (11.4)
Risk factors	
MSM	61 (34.7)
Heterosexual and multiple partners	59 (33.5)
Intravenous drug use	16 (9.1)
Unhoused	28 (15.9)
Comorbid conditions	
Hypertension	24 (13.6)
Diabetes	11 (6.3)
Cardiovascular disease	5 (2.8)
Chronic kidney disease	3 (1.7)
STI history	
Any STI	32 (18.2)
Chlamydia	7 (4)
Gonorrhea	14 (8)
Syphilis	15 (8.5)

Data shown as no. (%) unless otherwise noted.

Abbreviations: IQR, interquartile range; MSM, men who have sex with men; STI, sexually transmitted infection.

Of the 176 participants with a new HIV diagnosis, most (n = 117; 66.5%) were diagnosed through the BPA system only. Other common reasons for ED screening included high-risk behaviors (n = 27; 15.3%), concern for STI (n = 16; 9.1%), and presence of an opportunistic infection (n = 11; 6.3%). Most had an ED visit documented at any time in the Henry Ford Health EMR (n = 131; 74.4%) before their HIV diagnosis. Of the newly diagnosed patients, 69 (39.2%) were hospitalized and 61 (34.7%) had a concomitant STI diagnosis, most commonly syphilis in 21% (Table 3).

**Table 3.** Encounter Characteristics and Clinical Features of Patients Newly Diagnosed with HIV in the Emergency Department

Encounter and Clinical Features	Total No. (%) (N = 176)
Indication for ED HIV test	
Best practice alert only	117 (66.5)
STI screen	16 (9.1)
Pregnancy	2 (1.1)
Opportunistic infection	11 (6.3)
High-risk behavior	27 (15.3)
Multiple reasons	3 (1.7)
Medical history	
Prior HIV test	39 (22.2)
Prior ED visit	131 (74.4)
ED visit outcomes	
Hospitalized	69 (39.2)
Acute HIV	21 (11.9)
Concomitant STI diagnosed	
Any STI	61 (34.7)
Chlamydia	10 (5.7)
Gonorrhea	12 (6.8)
Syphilis	37 (21)

Abbreviations: ED, emergency department; STI, sexually transmitted infection.

After HIV diagnosis in the ED, approximately half of the patients (n = 82; 46.6%) attended their first scheduled ID clinic appointment, which was scheduled within 7 days of diagnosis or hospital discharge if the patient was admitted. Almost 60% (104/176) attended any ID clinic appointment within 90 days of diagnosis. For these 104 patients, the median number of days from diagnosis to first appointment was 13 days (IQR, 5–28). For the 107 patients who started ART, the median time to ART initiation from diagnosis was 9 days (IQR, 5–25). Slightly more patients initiated ART than attended postdiagnostic appointment (107 vs 104), as some patients were started on ART during hospitalization. For the 123 patients with evidence of baseline viral load testing, the median baseline viral load was 95 953 copies/mL. For the 119 patients who had evidence of immunologic testing, the median CD4 was 306 cells/mm^3^. Of those with available data, 51/77 patients (67.8%) had documented viral suppression within 3 months; 45/70 (71.1%) had viral suppression at 6 months, and 54/74 (72.9%) had viral suppression at 12 months of ART initiation (Table 4).

**Table 4.** Outcomes of Patients with HIV Infection Newly Diagnosed in the Emergency Department

Outcome	Value
Linked to medical care, no. (%)	
Attended first appointment (N = 176)	82 (46.6)
Attended any appointment^a,b^ (N = 176)	104 (59.1)
Time to medical care, median (IQR)	
Days to initial appointment^a,b^ (N = 104)	13 (5–28)
Days to ART initiation^b^ (N = 107)	9 (5–25)
Baseline laboratory values, median (IQR)	
Viral load, copies/mL (N = 123)	95 953 (22 889–631 859)
CD4 count, cells/mm^3^ (N = 119)	306 (164–572)
Viral suppression,^c^ no. (%)	
At 3 m (N = 77)	51 (66.2)
At 6 m (N = 70)	45 (64.3)
At 12 m (N = 74)	54 (73.0)

Abbreviations: ART, antiretroviral therapy; IQR, interquartile range.

^a^Appointment at Henry Ford Hospital only.

^b^Within 90 d.

^c^Viral suppression = viral load <30 copies/mL. Time points measured from ART initiation.

## DISCUSSION

After implementation of a nontargeted, opt-out HIV screening program in the Henry Ford Hospital ED, 176 patients were newly diagnosed with HIV infection over a 5-year period, yielding a new test-positivity rate of 0.23%. About two-thirds of the patients were diagnosed based on HIV testing triggered through the BPA system alone, underscoring the value of an automatic BPA system in the ED for detecting unrecognized HIV infections that might otherwise have been missed. More than half of the persons with HIV infection newly diagnosed in the ED attended a clinical care appointment within 2 weeks of diagnosis, with many subsequently initiating ART and attaining viral suppression, indicating that programs like ours can facilitate linkage to care and rapid treatment initiation.

Our BPA healthcare provider acceptance rate was high (72.4%), which is higher than general rates of BPA acceptance reported at 4% to 51% [7]. We attribute our BPA program's high uptake and acceptance to the close collaboration between the ED and ID teams. The ID team notified people of confirmed positive HIV results, which removed this task from the ED workflow and eliminated the need for ED clinicians to facilitate postdischarge management of newly diagnosed HIV infections. Importantly, our screening rates remained stable during the COVID-19 pandemic, highlighting the resilience of an opt-out, EMR-integrated screening model in the ED, even when the ED workflow is under stress. Our program demonstrates that an EMR-based, opt-out ED screening strategy is a feasible way to identify previously unrecognized HIV infections and facilitate timely linkage to care in an underserved urban population that relies heavily on the ED for basic care.

The new HIV test-positivity rate across the entire study period of 0.23% exceeded the 0.1% threshold recommended by the Centers for Disease Control and Prevention for routine, opt-out HIV screening to support cost effectiveness [1], suggesting that ED-based BPA screening is not only feasible but also may identify new diagnoses at rates well above the current benchmark. Also, our study revealed some key features of people with undetected HIV who required care in the ED. The median CD4^+^ count of our population was 306 cells/mm^3^ with low rates of opportunistic infections (6.3%), which together suggest that the BPA-based screening approach identified these patients early, before progression to advanced disease. Identifying HIV and initiating therapy before advanced disease is associated with improved long-term patient outcomes, further highlighting the value of ED screening programs that reduce missed opportunities for timely diagnosis within the acute care setting [8].

In our study, persons with newly identified HIV infection had a short median time to ART initiation, and patients were seen for an initial ID visit at a median time of only 13 days after diagnosis, which is within the US federal government's 2025 goal of 1 month [9]. We believe that the rapid linkage to care observed in our cohort reflects the structured collaboration between ED and ID teams, which is consistent with studies showing that active linkage strategies are significantly more effective than passive approaches [10]. The ID team's role in our system went beyond test disclosure and included other patient support tasks, such as assisting with appointment scheduling, maintaining contact until linkage to care was established, and providing case management services [8]. This collaborative, patient-centered approach is supported by prior evidence, including a randomized trial which showed that brief case management focused on identifying patient's individual strengths, reducing barriers, and accompanying persons to appointments was more effective than passive referral [11].

Rapid ART initiation after HIV diagnosis is endorsed by major US guidelines and represents a cornerstone approach to ending the HIV epidemic, as prompt treatment is associated with improved viral suppression, greater retention in care, and lower mortality [8]. In our study, patients who received rapid ART initiation showed sustained viral suppression over 12 months of follow-up, with suppression rates up to 73% in those who had data available. These viral suppression rates were consistent with the overall reported rates of 72% in Michigan in 2023 [12]. Data regarding viral suppression in patients newly diagnosed with HIV in the ED are limited, although rates in these populations tend to be lower than national rates because of greater levels of social vulnerability, homelessness, and other structural barriers [13]. The viral suppression rates observed in our study address an important gap in the literature regarding postdiagnosis outcomes in patients newly diagnosed with HIV in the ED.

Similar to the San Francisco RAPID program [14], our program reached populations disproportionately impacted by HIV, including a predominantly Black population, unhoused individuals, and those with substance use disorders. Because rapid ART initiation requires removal of structural barriers and is resource intensive, the ED provides a critical access point to engage high-risk populations who may otherwise have limited access to health care. Through close collaboration between ED and ID teams, our BPA system for ED HIV screening addressed these structural inequities and rapidly linked patients to care, advancing equitable access to timely HIV treatment.

### Limitations

Our study had several limitations. Given its retrospective design, patients diagnosed through ED screening may not reflect the overall demographics of the HIV epidemic. The MSM risk factor in our study was lower than the proportion of MSM accounting for incident HIV in Michigan [15]. We additionally could not reliably assess the reasons that providers opted out of BPA testing, and no data were available regarding the characteristics of patients who declined HIV testing. Data regarding ID clinic linkage to care, viral load suppression, and CD4^+^ count trends were unavailable for those who sought medical care outside of the Henry Ford Health system. There were 72 patients who did not have data regarding clinic appointment attendance, and we could not determine whether these individuals sought care at an outside clinic or did not pursue additional care at all. Michigan Department of Health and Human Services data from 2021 to 2024 for new HIV diagnoses made specifically at Henry Ford Health reported 6-month viral suppression at 78%, which is higher than our findings, indicating that a significant portion of individuals who followed up outside of Henry Ford Health were not captured in this study. Additionally, patients who died during the study period were included but did not have follow-up data.

The ART initiation was delayed in patients who were hospitalized with certain opportunistic infections, such as cryptococcal meningitis, which increased the median time to ART initiation in statistical analysis. The period of data collection overlapped with the onset of the COVID-19 pandemic, which may have negatively affected linkage-to-care efforts due to staffing challenges during peak periods of the pandemic. Lastly, although we believe that the success of linkage to care in our program was due to the close collaboration between the ED and ID teams, we do not have data from before implementation of the BPA program and therefore cannot formally assess the association between ED–ID collaboration and linkage to care.

### Conclusions

Our automated BPA-based, nontargeted, opt-out HIV screening program in the ED that embeds close ED and ID team collaboration may be a highly valuable and feasible model for identifying previously undiagnosed HIV in vulnerable populations, reducing missed opportunities for diagnosis in the acute care setting. Crucially, collaboration between ED and ID teams is essential for facilitating linkage to care and rapid ART initiation, which promotes viral suppression, improves patient outcomes, and reduces ongoing HIV transmission.
